# Functionally adaptive smart metasurface window with Weber beams for obstacle-avoiding communications in vehicle cabin

**DOI:** 10.1093/nsr/nwag083

**Published:** 2026-02-05

**Authors:** Shanwen Luo, Jun Xia, Yuxiang Wang, Ruizhe Jiang, Qinnan Xie, Gaiping Hao, Yu Luo, Tie Jun Cui, Jingjing Zhang

**Affiliations:** State Key Laboratory of Millimeter Waves, Southeast University, Nanjing 210096, China; Zhangjiang Laboratory, Shanghai 201210, China; State Key Laboratory of Millimeter Waves, Southeast University, Nanjing 210096, China; State Key Laboratory of Millimeter Waves, Southeast University, Nanjing 210096, China; State Key Laboratory of Millimeter Waves, Southeast University, Nanjing 210096, China; Zhangjiang Laboratory, Shanghai 201210, China; State Key Laboratory of Millimeter Waves, Southeast University, Nanjing 210096, China; State Key Laboratory of Millimeter Waves, Southeast University, Nanjing 210096, China; National Key Laboratory of Microwave Photonics, Nanjing University of Aeronautics and Astronautics, Nanjing 211106, China; State Key Laboratory of Millimeter Waves, Southeast University, Nanjing 210096, China; State Key Laboratory of Millimeter Waves, Southeast University, Nanjing 210096, China

**Keywords:** non-diffracting beams, metasurfaces, optical transparency, wireless communications, intra-vehicle network

## Abstract

The integration of non-diffracting beams with metasurfaces offers exceptional potential for advanced wave control, yet their practical deployment in communication systems, especially those requiring optical transparency, has remained largely unrealized. Here, we propose a transparent metasurface as a smart window that bridges this gap by generating self-accelerating Weber beams for robust in-vehicle wireless communications. Fabricated via a low-cost printed circuit board (PCB) process on flexible polyethylene terephthalate (PET) substrates, the smart window achieves 73.5% optical transmittance and efficient microwave cross-polarization conversion. Two beam types, i.e. orthogonal and semi-auto-focusing Weber beams, are tailored for lateral and rear windshield integration, both exhibiting self-bending and self-healing capabilities. In real-world vehicle cabin tests, the metasurface-enhanced system significantly improves the link reliability under physical obstruction, yielding an average enhanced power of 8.37 dB and reduced bit error rate below 1% with successful high-fidelity RGB image reconstruction. This work establishes a practical framework for transparent electromagnetic interfaces that dynamically reroute the wireless signals, with promising applications in smart vehicles, building-integrated communications, biomedical devices and industrial Internet of Things.

## Teaser

This work reports a transparent metasurface window generating self-healing Weber beams for robust wireless communications in obstructed vehicle cabins.

## INTRODUCTION

Metasurfaces, composed of subwavelength meta-atoms arranged in tailored configurations, have revolutionized electromagnetic (EM) wave control by enabling precise manipulation of amplitude, phase and polarization [1–11]. Recent progress in transparent fabrication techniques [12–20], particularly those employing indium tin oxide (ITO) [12,16,17] and metal meshes [18], further broadened the applicability of metasurfaces, allowing seamless integration into EM systems while maintaining high visible light transmittance. This synergy between optical transparency and wave manipulation has opened new possibilities in smart windows [21], augmented reality displays [22] and transparent antennas [23], where the dual requirements of optical clarity and high-performance wireless communication are essential.

Among the diverse application scenarios for transparent metasurfaces, vehicle windshields stand out as a particularly promising research domain with substantial practical implications. The rapid development of vehicle-to-everything (V2X) networks [24–26] and the vision of ubiquitous connectivity in sixth-generation (6G) wireless communication systems [27–29] have intensified the need for reliable in-cabin communications, yet the vehicle cabin presents unique communication challenges that are highly scenario-dependent [30–33]. On the one hand, the metallic body and complex interior components, such as headrests and rear displays, collectively create an enclosed and confined EM environment. On the other hand, the variable positions of multiple passengers introduce unpredictable signal-blocking effects on the signal transmission. Although reconfigurable intelligence surfaces (RISs) combined with multiple-input multiple-output (MIMO) systems offer a theoretical path to better coverage [34–36], their practical implementation is constrained by cost and integration complexity. To overcome the challenges posed by complex and cluttered transmission links, a key solution lies in an often-overlooked component: the vehicle windshield. As a natural interface between the external environment and cabin interior, the windshields offer inherent spatial advantages for deploying transparent metasurfaces. Moreover, serving as dual-purpose relays for both visible light and microwaves, windshield-integrated transparent metasurfaces can reconfigure the in-cabin wireless communication paradigm and its signal pathways. However, existing transparent metasurfaces designed for ordinary windows have largely been limited to generating focused beams [13,14,21,37,38]. Their effective gain is confined to a narrow spatial region, making them unsuitable to serve multiple passengers distributed throughout the cabin. Additionally, they lack the capability to circumvent obstacles such as seatback, headrests or passengers themselves—a fundamental requirement in real vehicle environments.

Non-diffracting beams (NDBs) provide a compelling alternative for obstacle-robust communications [39–44]. Generated by the interference of multiple plane waves with different propagation vectors, NDBs preserve their transverse intensity profile over extended distances [45,46]. Their intrinsic self-acceleration enables curved trajectories without external guidance, while their self-healing property allows reconstruction of the wavefront after obstruction. These features make NDBs ideally suited to cluttered settings like vehicle cabins, where conventional beams often fail to maintain reliable communication links. Several NDB families, such as Bessel beams [47,48], Airy beams [49–52], Mathieu beams [53] and Weber beams [54], have been studied across optics [50], terahertz [55] and microwaves [56], with demonstrations of self-bending [50,57] and self-recovery [58,59]. Nevertheless, their generation using transparent metasurfaces has not been explored, nor has their application to in-vehicle obstacle avoidance been proposed.

**Figure 1.**
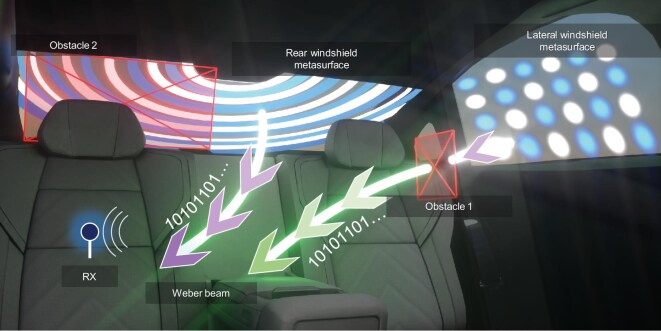
Schematic of windshield metasurfaces and Weber beams. The orthogonal and semi-auto-focusing distribution patterns are respectively implemented on the lateral and rear windshields to address diverse in-cabin scenarios.

In this work, we bridge these two frontiers by designing, fabricating and testing transparent metasurfaces that generate customized Weber beams for robust intra-vehicle communications. We introduce two distinct metasurface types: a lateral windshield metasurface (LWM) producing orthogonal Weber beams (OWBs); and a rear windshield metasurface (RWM) producing semi-auto-focusing Weber beams (SAFWBs), as shown in Fig. 1. Fabricated using cost-effective fine copper patterning on flexible polyethylene terephthalate (PET) substrates, these smart windows achieve an optimal balance between optical transparency (73.5% transmittance) and high microwave transmission efficiency (S_21_ = −1.9 dB). Through near-field measurements and in-vehicle wireless tests, we demonstrate reliable beam self-bending and self-recovery around metallic obstacles, yielding an average received power gain of 8.37 dB and bit error rate below 1% in obstructed scenarios. This study establishes a practical pathway for embedding intelligent wave control into transparent surfaces, with implications beyond automobiles in smart infrastructure, biomedical sensing and industrial Internet of Things (IoT).

## RESULTS

### Theory and fabrication

An in-cabin environment analysis was conducted to determine the propagation path of the Weber beam and the resulting field distribution on the windshield metasurface. This investigation led to the design of the LWM and RWM, which target the two primary modulation effects identified: lateral and rear field distribution. The Weber beams for these metasurfaces are then custom-designed based on application-specific needs, generating their unique field distribution profiles.

**Figure 2.**
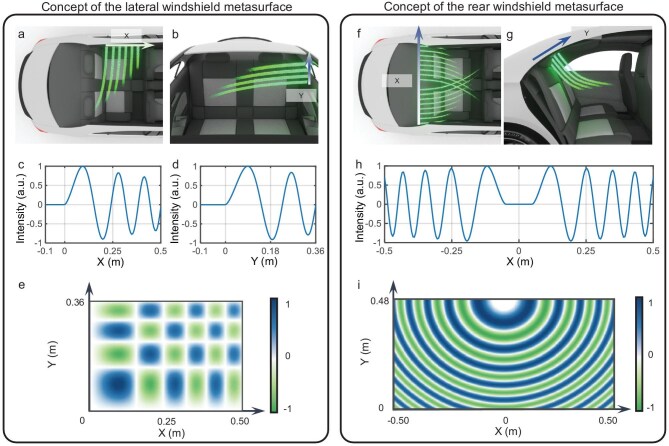
Integration of windshield metasurface design principles with Weber functions for EM wave manipulations. The (a) *x*-sectional and (b) *y*-sectional distributions of the EM waves excited by the LWM. The (c) *x*-direction and (d) *y*-direction 1D Weber functions employed in the LWM. (e) The normalized electric-field intensity distribution of the LWM. The (f) *x*-sectional and (g) *y*-sectional distributions of the EM waves excited by the RWM. (h) The *x*-direction 1D Weber function employed in the RWM. (i) The normalized electric field intensity distribution of the RWM.

For LWM, the cross-sectional profiles along the *x*- and *y*-axes are shown in Fig. 2a and b. In the *x* dimension, given the posterior positioning of the passenger electronic device activity zone relative to the LWM, the Weber beams at this cross-section exhibit accelerated propagation characteristics toward the rear of the vehicle. Similarly, in the *y* dimension, the activity area lies below the LWM, requiring downward beam acceleration. The self-healing property inherent to Weber beams enables signal transmission through localized obstructions near the windshield via constructive interference and sidelobe superposition, thereby facilitating robust connectivity to passengers on the opposite side. Based on the typical sedan cabin and the lateral windshield dimensions, we derived the 1D Weber functions in *x* and *y* directions as:


(1) \begin{eqnarray*}
{W}_x = {e}^{ - ax}\sin \left(\frac{4}{3}{b}^{\frac{1}{2}}k{x}^{\frac{3}{2}} \right)\frac{1}{g},
\end{eqnarray*}

(2) \begin{eqnarray*}
{W}_y = {e}^{ - ay}\sin \left(\frac{4}{3}{b}^{\frac{1}{2}}k{y}^{\frac{3}{2}} \right)\frac{1}{g},
\end{eqnarray*}

where the decay factor is *a* = 1, the scaling factor is *b* = 0.15 and the correction factor is *g* = 0.91 for the *x* direction, and *b* = 0.18 for the *y* direction. The wavenumber is given by *k* = 2π*f*/*c*, with the frequency *f* = 4.9 GHz and *c* is the speed of light. The corresponding 1D Weber functions are plotted in Fig. 2c and d. Multiplying the 1D Weber functions in the *x* and *y* directions yields the OWB field distribution presented in Fig. 2e. Note that the sign of the amplitude indicates a 180° phase difference.

The RWM presents distinct EM characteristics and communication requirements compared to the LWM. As shown in Fig. 2f, although the rear windshield has a larger surface area, the space behind the rear seats often contains objects that cause significant EM interference. To address this, we leverage the self-accelerating property of Weber beams to design two counter-propagating 1D Weber beams along the *x* dimension. In unobstructed scenarios, these beams focus energy from windshield edges into the electronic device activity zone via main lobe convergence. When obstacles are present (e.g. metallic objects behind headrests), the self-accelerating beam from the unobstructed side maintains power delivery to occluded receivers. Analysis in the *y* dimension in Fig. 2g indicates that the signal transmission from the rear windshield to the activity zone requires a curved beam trajectory, which is a behavior intrinsically matched to the self-accelerating nature of Weber beams. Consequently, we propose an SAFWB for the RWM, integrating properties from both dimensions. Its beam function, shown in Fig. 2h, comprises two symmetric Weber functions with an attenuation factor of *a* = 0.5 and a scaling factor of *b* = 0.3. The field distribution in Fig. 2i is obtained by sweeping the Weber function in Fig. 2h about point (*x* = 0, *y* = 0.48), with intentional compression in the *y* dimension to optimize the beam curvature through the lobe count adjustment. The dimensions of the SAFWB can be customized according to the rear windshield size of specific vehicle models.

**Figure 3.**
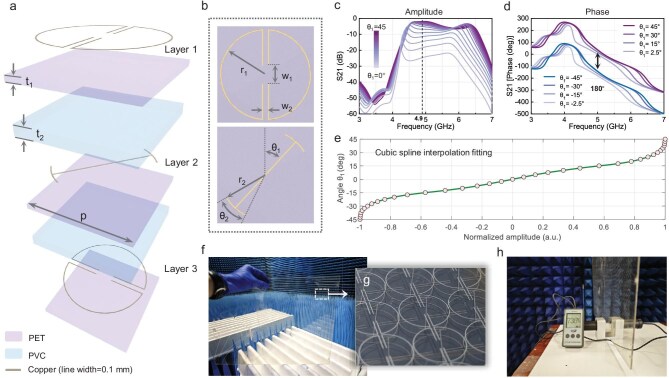
Fabrication process from unit cell design to metasurface realization. (a) Exploded view of unit, with *p* = 9.6 mm, *t*_1_ = 0.125 mm, *t*_2_ = 1.5 mm. (b) Top view of layer 1 and layer 2 of the unit, with *r*_1_ = *r*_2_ = 4.6 mm, *w*_1_ = 1.9 mm, *w*_2_ = 0.46 mm, *θ*_2_ = 33°. (c) Amplitude of the cross-polarized transmission coefficient (S21) as a function of the rotation angle *θ*_1_. (d) Phase difference between configurations with positive and negative *θ*_1_ angles. (e) Cubic spline interpolation relating the angle *θ*_1_ to the S21 amplitude at 4.9 GHz. (f) Photograph of the fabricated lateral windshield metasurface sample. (g) Close-up view of the fabricated unit cells. (h) Optical transmittance test of the metamaterial sample.

The unit cell structure of the windshield metasurface, as shown in Fig. 3a and b, consists of three precision copper line layers. Each copper layer is deposited on an ultra-thin PET substrate, with two polyvinyl chloride (PVC) interlayers inserted between the metallic layers to enhance EM coupling. The total thickness of the unit is 3.375 mm. In terms of material selection, while transparent conductive oxides (TCOs) such as ITO offer superior optical transmittance, their inherent sheet resistance can introduce non-negligible ohmic losses in high-efficiency microwave applications. Conversely, although high-resolution metal mesh structures provide excellent conductivity, their customized prototyping costs can be prohibitive for rapid experimental iterations. In this proof-of-concept study, we utilize a direct-etched copper-clad PET substrate. This approach offers a pragmatic balance, providing exceptional electrical conductivity necessary for precise Weber beam synthesis while maintaining cost-effectiveness for laboratory-scale fabrication, despite the trade-off in visible aesthetic appeal, which could be further optimized for mass production. Although conventional printed circuit board (PCB) technology offers a good compromise between conductivity and cost-effectiveness, the use of large-area metallic materials tends to reduce optical transparency. To resolve this trade-off, we designed the unit cell with 0.1 mm linewidth copper lines, achieving effective EM wave manipulation while maintaining high optical transparency. The unit period *p* is set to 9.6 mm (0.16 λ₀).

The physical realization of the target Weber-beam profiles requires both continuous amplitude modulation and 1-bit phase control. To satisfy these dual requirements, we employ the Pancharatnam–Berry (PB) phase principles by strategically rotating *θ*_1_ of the meta-atoms in layer 2. As displayed in Fig. 3c, progressively rotating *θ*_1_ from 0° to 45° increases the cross-polarized S21 response at 4.9 GHz from −30 to −1.9 dB. A similar amplitude variation is observed in the *θ₁* range from −45° to 0°. Meanwhile, Fig. 3d confirms a consistent 180° phase difference between symmetrically rotated domains (i.e. +*θ*_1_ and −*θ*_1_), thereby establishing the required binary phase shift.

The metasurface fabrication requires mapping unit cells with specific *θ*_1_ values according to the target field distribution shown in Fig. 2e and i. First, we extract the S21 data at 4.9 GHz from the *θ*_1_ parametric sweep and normalize the values. A cubic spline interpolation is then applied to establish a continuous function mapping amplitude to *θ*_1_, as shown in Fig. 3e. Note that the sign of the amplitude reflects the 180° phase difference. This approach simplifies the unit layout process by linking it directly to the amplitude distribution. The rotation angle *θ*_1_ for each position is determined by mapping the field amplitude values via the interpolation function. The fabricated metasurface sample and a close-up view of its unit cells are presented in Fig. 3f and g, demonstrating high optical transparency. Measured results in Fig. 3h confirm an optical transmittance of 73.5%, which meets standard automotive windshield requirements.

### Near-field measurements

**Figure 4.**
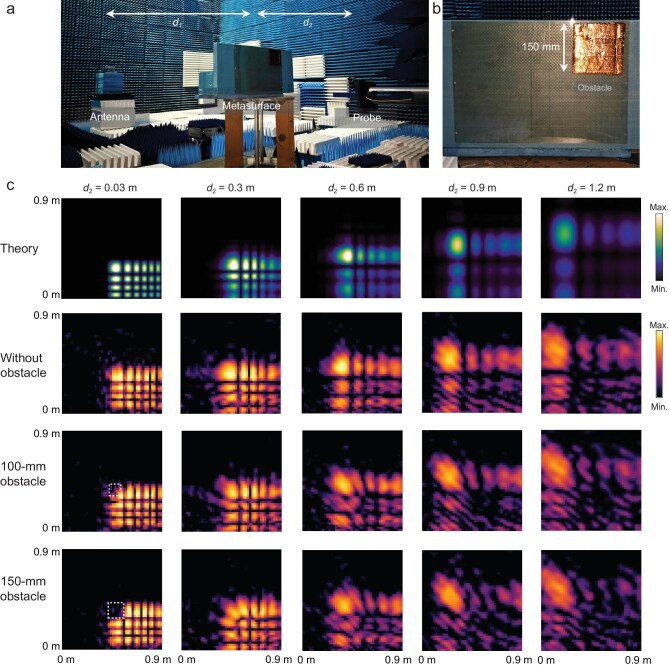
OWB near-field characterization. (a) Experimental setup for the near-field measurements. (b) The size and location of the metallic obstacle. (c) Theoretical and measured near-field distributions of orthogonal Weber beams along the propagation direction.

To characterize the OWB generated by the LWM, near-field measurements were conducted in a microwave anechoic chamber, as depicted in Fig. 4a and b. The distance between the antenna and the metasurface, *d*_1_, was fixed at 3.5 m. By varying the distance between the probe and the metasurface, *d*_2_, we acquired cross-sectional near-field distributions of the OWB at sequential propagation planes. A square-shaped metallic obstacle was positioned at the corner to block the main lobe. The top row of Fig. 4c displays the theoretically calculated near-field distributions along the propagation path, obtained using the Rayleigh–Sommerfeld diffraction integral:


(3) \begin{eqnarray*}
U(x,y,z) = \frac{1}{{i\lambda }}\int\!\!\int {{U}_0({x}_0,{y}_0)\frac{{{e}^{ikr}}}{r}K(\theta ){\rm d}x}{\rm d}y,
\end{eqnarray*}

where $r = \sqrt {{{(x - {x}_0)}}^2 + {{(y - {y}_0)}}^2 + {z}^2} $. The maximum propagation distance for both simulations and measurements was set to 1.2 m, corresponding to the width of a typical vehicle rear cabin. The second row of Fig. 4c presents measured results without obstacles, showing good agreement with theoretical predictions. Within 0.6 m, the OWB maintains consistent main lobe width and energy intensity, demonstrating its non-diffracting nature. Beyond this distance, side lobe energy gradually merges into the main lobe, leading to angular acceleration. The third row shows the case with a 100 mm metallic obstacle placed at the origin of the main lobe. Near-field scans confirm initial lobe disruption, though the beam recovers by 0.3 m due to self-healing via sidelobe interference. When the obstacle size is increased to 150 mm (bottom row), the main lobe is initially fully blocked but completely recovers by 0.6 m, demonstrating effective self-reconstruction.

**Figure 5.**
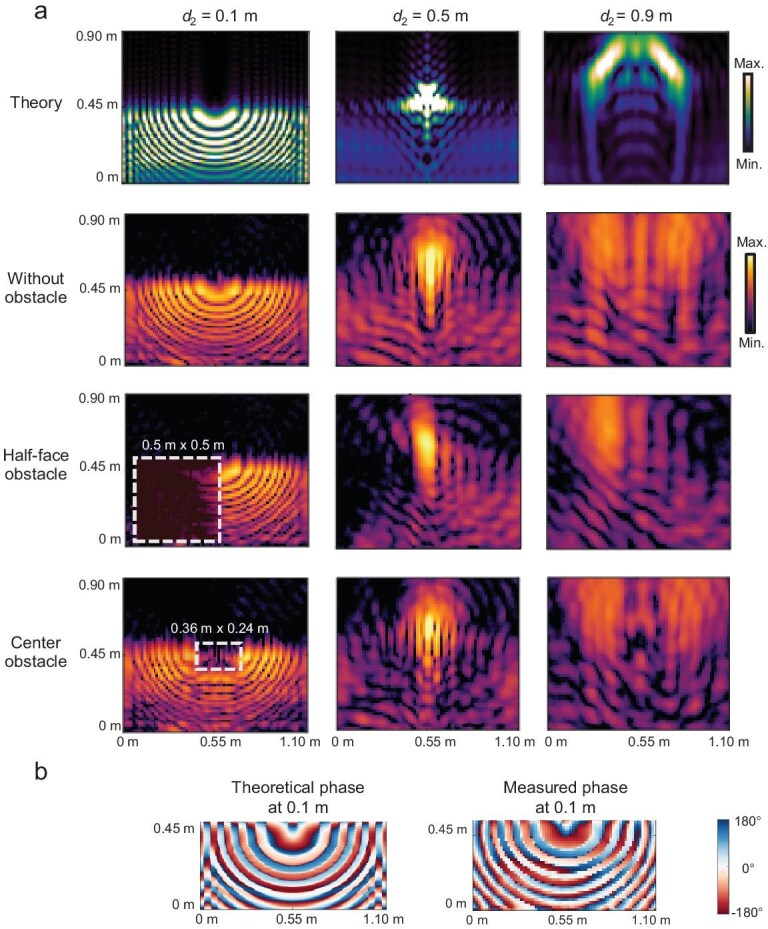
SAFWB near-field characterization. (a) Theoretical and measured near-field distributions of the self-accelerating folded Weber beam along the propagation direction. (b) Corresponding theoretical and measured phase distributions of the rear windshield metasurface at a propagation distance of 0.1 m.

Under identical anechoic chamber conditions, we performed near-field characterization of the RWM, with the results shown in Fig. 5. The SAFWB is constructed by sweeping a 1D Weber function. This design leverages the self-accelerating property of Weber beams to circumvent obstacles, particularly suited to the spatial constraints of rear windows. From a functional perspective, energy from the side lobes progressively converges into the main lobe. When numerous 1D Weber functions are arranged in a semi-circular configuration through sweeping, the peripheral side lobes converge at an off-axis focal point before propagating along two diagonal paths, as shown in rows 1–2 of Fig. 5a. The measured propagation trajectories match well with theoretical predictions.

In row 3 of Fig. 5a, a half-face metallic obstacle covering 50% of the RWM causes the wave energy from the bottom-right region to progressively shift toward the top-left quadrant with increasing propagation distance, confirming the self-accelerating behavior and cross-propagation behavior of the SAFWB. Row 4 shows the case with a central obstacle, and the striking resemblance between the obstructed (row 4) and the unobstructed (row 2) cases clearly validates the self-healing capability of the beam. Phase measurements in Fig. 5b further confirm the theoretical 180° phase alternation between adjacent lobes.

### Wireless communication tests

**Figure 6.**
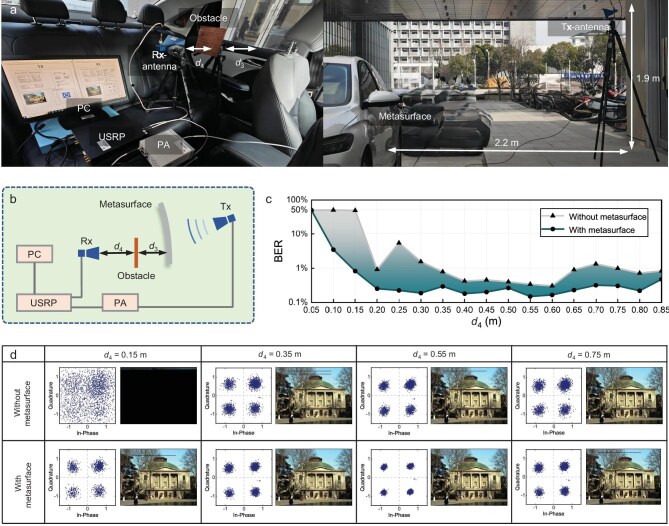
Wireless communication performance of the LWM. (a) Photographs of the in-vehicle scenario and the test setup. (b) Block diagram of the wireless communication system. (c) Measured BER comparison with and without the LWM. (d) Constellation diagrams and corresponding reconstructed images at selected transmission distances.

To evaluate the practical performance of the metasurface in wireless communications, we constructed an in-vehicle test platform for the LWM, as illustrated in Fig. 6a, with the corresponding system schematic shown in Fig. 6b. This wireless communication test involved transmitting red/green/blue (RGB) images to evaluate receiver performance through bit error rate (BER), constellation diagrams and reconstructed image quality. The setup employed a universal software radio peripheral (USRP) connected to horn antennas as the transmitter and receiver, with the LWM serving as a microwave relay that converts 4.9 GHz incident waves into an OWB to bypass metallic obstacles. The information bit rate was set to 2 Mbps. Digital bitstreams derived from RGB images underwent channel coding before being modulated via quadrature phase-shift keying (QPSK) and orthogonal frequency-division multiplexing (OFDM). The resulting in-phase and quadrature (IQ) data were then processed by the USRP’s radio frequency (RF) front-end for digital-to-analog conversion, amplified through a power amplifier, and transmitted. The signals propagating through the LWM-enabled path were subsequently demodulated through the inverse process to reconstruct the original RGB images at the receiver.

Quantitative analysis of the obstacle-avoiding communication is presented in Fig. 6c and d. A square metallic obstacle of 0.2 m × 0.2 m was positioned at the OWB main lobe at a fixed distance of *d*_3_ = 0.2 m from the LWM. The separation *d*_4_ between the obstacle and the receiving antenna was increased from 0.05 to 0.85 m in 0.05 m increments. BERs were measured both with and without the metasurface. At *d*_4_ = 0.05 m, both configurations exhibited 50% BER, indicating severe signal degradation due to obstacle-induced interference. When *d*_4_ reached 0.15 m, the metasurface-enabled system restored the obstructed main lobe signal, reducing the BER to below 1% with successful RGB image reconstruction. As *d*_4_ was extended to 0.55 m, both systems achieved minimum BER, though constellation diagrams showed tighter symbol clustering with the metasurface. Beyond 0.55 m, the BER increased in both cases, yet consistently remained at lower values with the metasurface across the extended propagation range.

**Figure 7.**
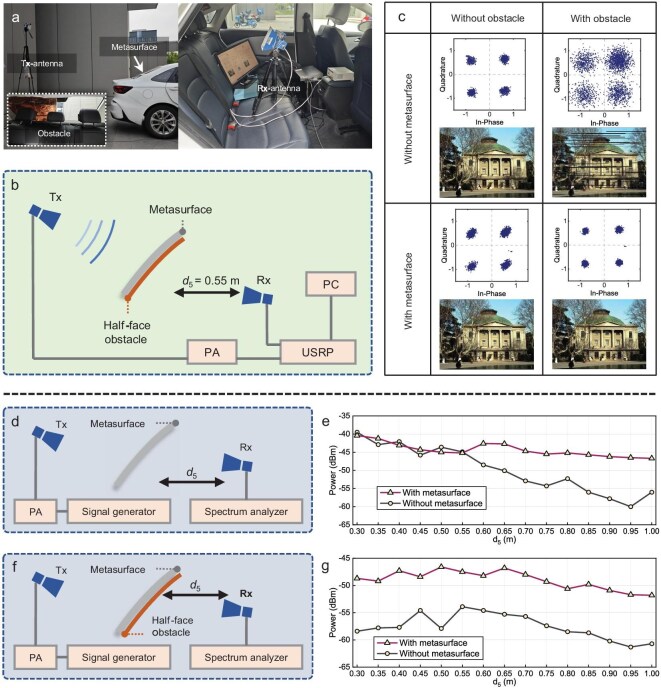
Wireless communication tests on the RWM. (a) Photos of the testing setups and scenes. (b) Logical framework for the wireless communication tests. (c) Constellation diagrams and corresponding recovered pictures under various conditions of loading RWM and obstacle. (d) Experimental framework of signal power measurement without obstacle. (e) Comparison of received signal power with and without the loading of RWM in the absence of obstacle. (f) Experimental framework of signal power measurement with obstacle. (g) Comparison of received signal power with and without the loading of RWM in the presence of obstacle.

Identical equipment configurations were employed for RGB image transmission tests with the RWM, as shown in Fig. 7a and b. A 4.9 GHz EM wave was incident on the RWM from the transmitting antenna, with the receiving antenna positioned at distance *d*_5_ from the seat headrest and located on the same side as potential obstacles. In the absence of obstacles, the receiver successfully decoded the transmitted images and produced clear constellation diagrams, regardless of whether the metasurface was used, as shown in the first column of Fig. 7c. However, when a half-face metallic obstacle was placed on the rear windshield without RWM assistance, the constellation diagrams deteriorated significantly and the receiver failed to recover valid images. Implementation of the RWM substantially improved both constellation diagram integrity and image reconstruction quality.

To investigate the signal enhancement and obstacle-avoiding capabilities of the SAFWB, we performed additional received signal power measurements under the same test configurations. As illustrated in Fig. 7d, a signal generator and power amplifier energized the transmitting antenna, while a spectrum analyzer measured the received power. First, without obstacles, the received power was recorded as the distance between the receiver and the headrest (*d₅*) was incrementally increased. The results in Fig. 7e show that the RWM had negligible influence on received power within the range of 0.30–0.55 m. Beyond *d₅* = 0.55 m, however, the extended-range enhancement provided by the SAFWB became evident, with a progressively widening power advantage over the system without the metasurface. When a half-face metallic obstacle was introduced on the receiver-side rear windshield, a significant difference in received power was observed, as shown in Fig. 7d and f. Across the range from 0.30 to 1.00 m, the RWM provided an average received power gain of 8.37 dB. This improvement originates from the efficient utilization of peripheral side-lobe energy by the SAFWB. Aiming for a more thorough demonstration of the in-cabin multi-user/multi-path environment, we conducted additional multi-connectivity experiments on the lateral and rear windshield, as detailed in Note 10 of the Supplementary data.

The selection of 4.9 GHz as the operating frequency is driven by both technical advantages and regulatory trends. This frequency falls in the NR N79 band of 5G wireless communications, an emerging mid-band spectrum that strikes a balance between the wide-area coverage and high-speed data transmission. Unlike the heavily researched N78 band, N79 is tailored for high-capacity hotspots and industrial private networks, as designated by regulatory bodies. In terms of the EM environment, the 4.9 GHz band offers a cleaner spectrum with broader bandwidth for vehicular applications than the interference-prone 2.4 GHz industrial, scientific and medical (ISM) band. Moreover, considering that the official cellular V2X (C-V2X) frequency is allocated around 5.9 GHz, the proposed metasurface design at 4.9 GHz provides a scalable framework that can be readily adapted for future intelligent transportation systems.

The practical implementation of the proposed metasurface window is based on a multilayer integration strategy designed for the automotive environment. The functional core consists of three-layer copper-clad PET films, which exhibit high thermal endurance (up to 200°C) and superior mechanical flexibility due to the ultra-thin nature of the substrate (0.125 mm). While PVC spacers were used in our prototype for proof-of-concept validation to maintain requisite interlayer separation, industrial-scale implementation would involve embedding these conductive layers directly within the interlayers of laminated safety glass, e.g. using polyvinyl butyral (PVB) or ethylene vinyl acetate (EVA) films. This encapsulation strategy offers a dual advantage; it ensures the structural integrity of the window while hermetically shielding the metallic elements from atmospheric exposure, thereby preventing oxidation and long-term degradation.

Finally, we compare our work with previous studies on the transparent metasurfaces and NDB metasurfaces in Table 1. Our design distinguishes itself in several key aspects; it leverages a low-cost, well-established PCB process on copper-clad PET substrates, achieves high transmittance in both microwave and optical spectra, and introduces a novel NDB mode tailored for in-vehicle wireless environments. More importantly, the system’s capability to circumvent the obstacles and sustain communication has been quantitatively verified under realistic conditions, demonstrating reliable performance facilitated by tailored Weber beams. By harmonizing performance, fabricability and practicality, this work bridges conceptual innovation with implementable design, moving metasurface technology closer to real-world vehicular applications.

**Table 1.** Comparison of different works on transparent metasurfaces and NDB metasurfaces.

Ref.	Fabrication technology	Operating frequency	Energy efficiency^a^	Visible light transmittance (%)	Generated specific beams	Verification of obstacle avoidance communication	Application scenarios
[13]	ITO depositing	5.8 GHz	S11 −1 dB	63	Focusing beam	N/A	N/A
[21]	ITO depositing	3.5 GHz	S21 −1.94 dB	N/A	Focusing beam	N/A	Window
[37]	Silver etching	38.5 GHz	S21 −0.9 dB	N/A	Focusing beam	N/A	Vehicle cabin
[60]	Quartz + ITO depositing	27.9 GHz	S21 −0.96 dB	51.7	N/A	N/A	N/A
[41]	3D printing	130 GHz	N/A	Not transparent	Bessel beam	Quantitative verification	N/A
[42]	3D printing	3.17 kHz	N/A	Not transparent	Airy beam	Qualitative verification	N/A
[43]	3D printing	200 GHz	S21 −1 dB	Not transparent	Airy beam	Qualitative verification	N/A
[44]	TiO_2_ depositing	580 THz	PCE 77.5%	N/A	Airy beam	Quantitative verification	Underwater
This work	PCB	4.9 GHz	S21 −1.9 dB	73.5	Weber beam	Quantitative verification	Vehicle cabin

PCE, polarization conversion efficiency.

a Characterizes the maximum energy rate of the incident EM wave transformed into the specific beams after passing through the sample, such as scattering parameters, i.e. S21 for transmission-type samples and S11 for reflection-type samples.

## CONCLUSIONS

In summary, we developed a transparent metasurface platform that integrates non-diffracting Weber beams with optical transparency to overcome signal obstruction in complex wireless environments. Using a PB phase-based meta-atom design and low-cost fabrication, we realized a windshield-compatible metasurface that maintains high optical clarity while enabling tailored self-accelerating and self-healing beam propagation. These beams reliably steer signals around obstacles, as validated through in-vehicle wireless tests. Beyond automotive applications, this work provides a scalable framework for embedding intelligent wave–control interfaces into transparent surfaces from architectural windows to wearable devices, paving the way for dynamically reconfigurable signal management in future smart EM environments.

## METHODS

### Interlayer bonding method for metasurfaces

To bond the successive dielectric layers of the metasurface while preserving high optical transparency, an ultraviolet (UV)-curable adhesive was used. The adhesive was applied uniformly between the PVC and PET layers. The layers were then aligned, pressed together and cured under high-intensity UV light to form a robust, transparent bond across the full aperture of the metasurface. The transmittance measurements presented in Fig. 3h were obtained using an LS116 spectrophotometer.

### Near-field experimental setup

The near-field measurements employed a setup consisting of a horn antenna (transmitter) and a field probe (receiver), both connected to a vector network analyzer (VNA). The metasurface was positioned between the horn and the probe, aligned with the horn’s central axis. A 2D scanning system translated the probe across a plane parallel to the metasurface. Field profiles were acquired through a discrete spatial scan with a uniform sampling interval of 20 mm. The setup used for Fig. 4a includes a horn antenna (3.9–6 GHz), a waveguide probe (3.94–5.99 GHz) and a Rohde & Schwarz ZVA50 VNA.

### Wireless communication experimental setup

The wireless communication system was implemented in LabVIEW and executed on USRP hardware for signal processing and transmission. As shown in Fig. 6a, the communication tests utilized two horn antennas, a Ceyear 80246A power amplifier and a USRP-LW X310 software-defined radio. For the signal power measurements in Fig. 7d, a Ceyear 80246A power amplifier, an Agilent E8267D signal generator and an Agilent N9010A spectrum analyzer were used.

## Supplementary Material

nwag083_Supplemental_File

## Data Availability

The data that support the plots within this paper and other findings of this study are available from the corresponding author upon reasonable request.
