# Supplementary material for: Functionally adaptive smart metasurface window with Weber beams for obstacle-avoiding communications in vehicle cabin
Source: Natl Sci Rev. 2026 Feb 5;13(6):nwag083. doi: 10.1093/nsr/nwag083 (PMC13017836; doi:10.1093/nsr/nwag083)
Supplement: nwag083_Supplemental_File [file nwag083_supplemental_file.pdf]

Supplementary Information

# Functionally Adaptive Smart Metasurface Window with Weber Beams for Obstacle-Avoiding Communications in Vehicle Cabin

Shanwen Luo[1,2], Jun Xia[1], Yuxiang Wang[1], Ruizhe Jiang[1,2], Qinnan Xie[1], Gaiping Hao[1], Yu Luo[3*], Tie Jun Cui[1*], Jingjing Zhang[1*]

1 State Key Laboratory of Millimeter Waves, Southeast University, Nanjing 210096, China

2 Zhangjiang Laboratory, Shanghai 201210, China

3 National Key Laboratory of Microwave Photonics, Nanjing University of Aeronautics and Astronautics, Nanjing 211106, China.

*E-mails: zhangjingjing@seu.edu.cn, tjcui@seu.edu.cn, yu.luo@nuaa.edu.cn*

Supplementary Note 1. Bending conformal simulation……………………………………...2

Supplementary Note 2. Different distribution of RWM……………………………………..4

Supplementary Note 3. Detailed characterization of SAFWB propagation space………..……6

Supplementary Note 4. Schematic diagram of y-axis compression of SAFWB……………….8

Supplementary Note 5. Experimental and theoretical simulation results of self-accelerating and self-healing characteristics of SAFWB…………………………………………………………9

Supplementary Note 6. Comparison between Airy beam and Weber beam………………….11

Supplementary Note 7. Comparison of theoretical simulation and measured phase results of OWB ……………………………………………………………………………………….13

Supplementary Note 8. Interpolation fitting process………………………………………….14

Supplementary Note 9. Relative bandwidth…………………………………………………..15

Supplementary Note 10. Multi-user communication scenario………………………………..17

Supplementary Note 11. Communication scenario with passenger…………………………..19

Supplementary Note 12. The impact of obstacle size on communication…………………….21

## Supplementary Note 1. Bending conformal simulation

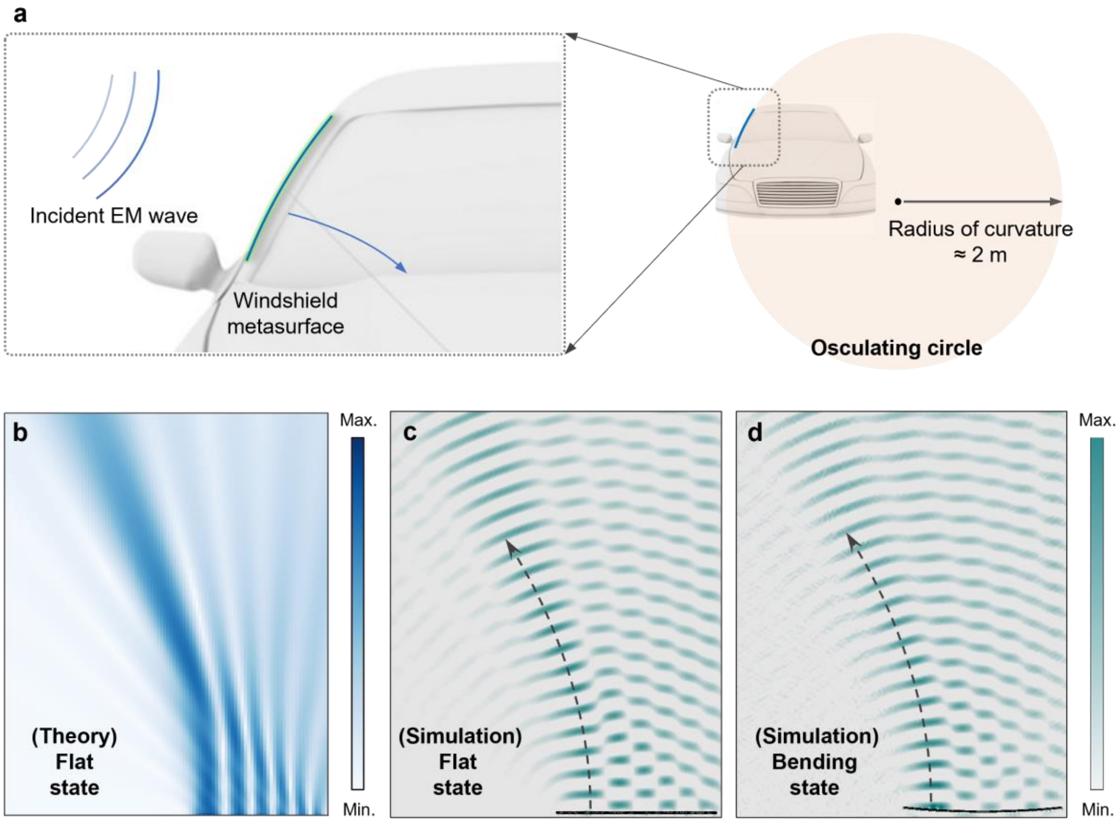


**Fig. S1.** Diffraction analysis of conformal Weber beams on curved windshield surfaces.

Within the theoretical framework of this article, we analyze the diffraction characteristics of Weber beams under idealized conditions. However, in practical automotive environments, vehicle windshields deviate from planar geometry, exhibiting curvature with specific radius. As documented in established automotive industry standards [1-3], the curvature radius of sedan lateral windshield typically approximates 2 m, illustrated in Fig. S1a. As illustrated in Fig. S1b, we theoretically calculate the two-dimensional diffraction pattern of the x-dimensional 1D Weber beam in Fig. 2c. Wherein, the 2D Rayleigh-Sommerfeld diffraction integral formula used is as follows

$$U(x,z)=\frac{1}{i\lambda}\int U_0(x_0)\frac{e^{ikr}}{r}K(\theta)dx\,, \tag{S1}$$

where $r=\sqrt{(x-x_0)^2+z^2}$. Furthermore, we construct the corresponding 1D array in CST Studio Suite for full-wave simulation, employing plane wave excitation. As demonstrated in Fig. S1c, the resulting diffraction pattern exhibits excellent agreement with theoretical predictions in terms of beam propagation characteristics. For comparative benchmarking, we implemented conformal bending of the 1D array to match the specified automotive curvature (radius $\approx$2m)

in simulations. The resulting Weber beam diffraction pattern closely aligns with the ideal case, validating the metasurface's adaptability to practical automotive integration scenarios.

## REFERENCES

1. Gao DW, Gao YK, Zhou XY, Liu HL. Automotive door glass and guide rail with drum surface. *J Tongji Univ, Nat Sci* 2012; **40**: 92-7.
2. Lei YC, Zhang P, Chen SC, Yi TS. Torus surface fitting for dual curvature car door glass. *Automot Eng* 2005; **27**: 623.
3. Gao YK, Zhao Y, Peng HD. Design method for car door glass with torus surface. *Automot Eng* 2005; **27**: 483.

## Supplementary Note 2. Different distribution of RWM

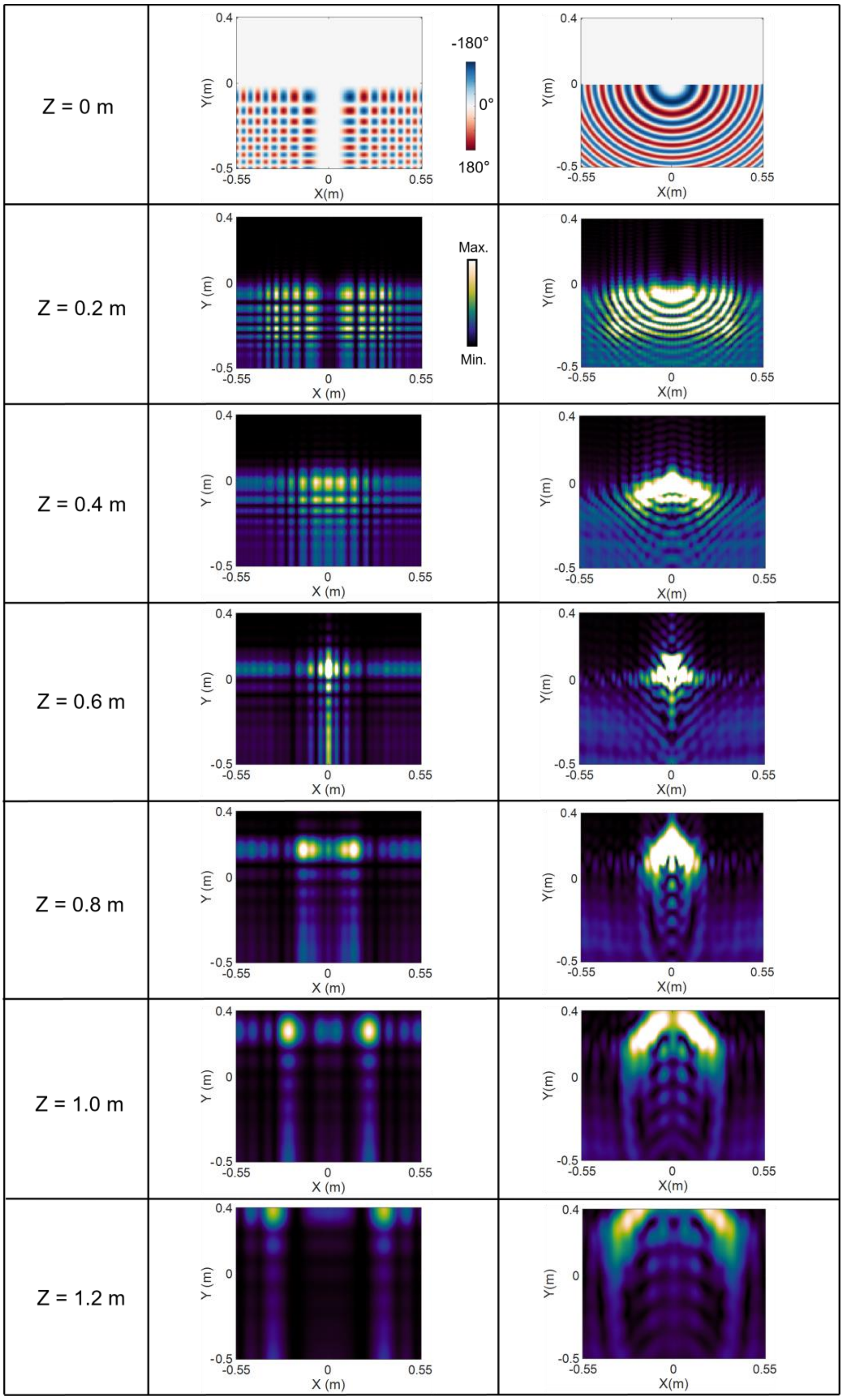


**Fig. S2.** Spatially calculated interface distributions along the propagation direction for both orthogonal mode and SAFWB mode of the RWM.

The right column in Fig. S2 is the metasurface distribution mode adopted for the rear windshield in this paper. Some readers may naturally wonder why the SAFWB mode was implemented for the RWM, rather than continuing with the orthogonal Weber beam scheme successfully used in LWM. The configuration strategy is motivated by three considerations. Firstly, SAFWB has stronger energy than the orthogonal mode in left column of Fig.S2. The distinction originates from beam formation principles. In the orthogonal mode, intensity is exponentially attenuated by the Weber function along both transverse dimensions. Conversely, the SAFWB is generated by sweeping a 1D Weber function, thus experiencing exponential decay in only one dimension. Secondly, SAFWB has a higher utilization rate of the surface area of the metasurface. It can be clearly seen from the comparison diagram of Z = 0 m that the void area of the orthogonal mode is larger than that of SAFWB. This retention of the spatial gap is necessitated by the physical separation between the rear windshield and back seats, as illustrated in Fig. 2f. To ensure focal-point convergence of beam energy on passengers, an intentional offset must be introduced between the two 1D Weber beams. Thirdly, In the propagation, the signal coverage cross section of SAFWB is larger. It can be clearly seen from the three comparisons of Z = 0.8 m, 1.0 m, and 1.2 m that the signal coverage cross section of SAFWB is larger than that of the orthogonal mode.

## Supplementary Note 3. Detailed characterization of SAFWB propagation space

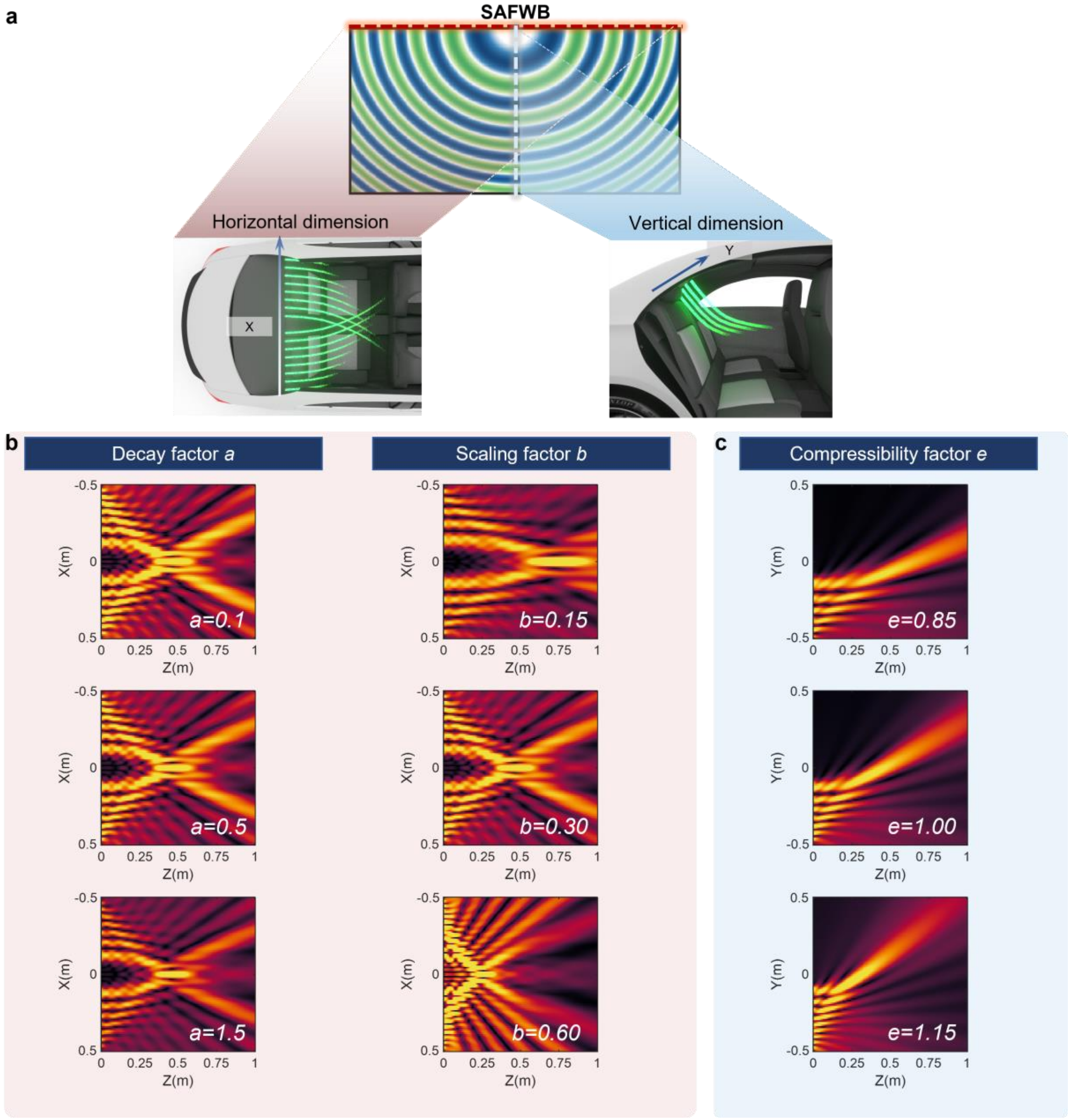


**Fig. S3.1.** (a) The initial energy distribution of SAFWB. Schematic diagram of horizontal dimension and longitudinal dimension of beam. (b) The influence of decay factor *a* and scaling factor *b* on the energy distribution of horizontal dimension. (c) Influence of compressibility factor *e* on energy distribution of vertical dimension.

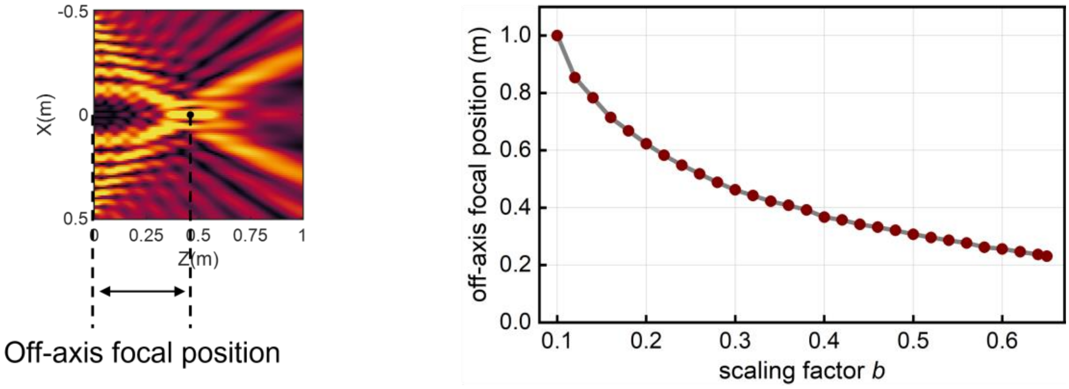


**Fig. S3.2.** Impact of the scaling factor *b* on the modulation of the SAFWB's off-axis focal position.

We analyzed the correlation between the selected Weber parameters, the sweep geometry, and the resulting off-axis focal location and beam propagation paths. This analysis is presented in Fig. S3.1. We conduct numerical simulation analysis of SAFWB propagation space in horizontal and vertical dimensions. The decay factor *a* directly determines the magnitude of the beam energy, while the scaling factor *b* serves as a key parameter for regulating the focal position of the beam. Specifically, a decrease in factor *b* results in the focal spot shifting further away, as shown in Fig. S3.2. The two focused beams located behind the main focal spot can also be modulated via factor *b*.

Regarding how to modulate the key parameters to adapt to different vehicle interiors, this can be understood through the analysis of the different dimensional beam cross-sections shown in Fig. S3.1. In the horizontal dimension, the scaling factor *b* can be used to tune the bending angle of the two Weber beams to accommodate varying seat widths and depths. If the vehicle features different rear windshield inclination angles, the beam curvature can be adjusted by tuning the compression factor *e* to ensure smooth propagation of the beam into the core region.

## Supplementary Note 4. Schematic diagram of y-axis compression of SAFWB

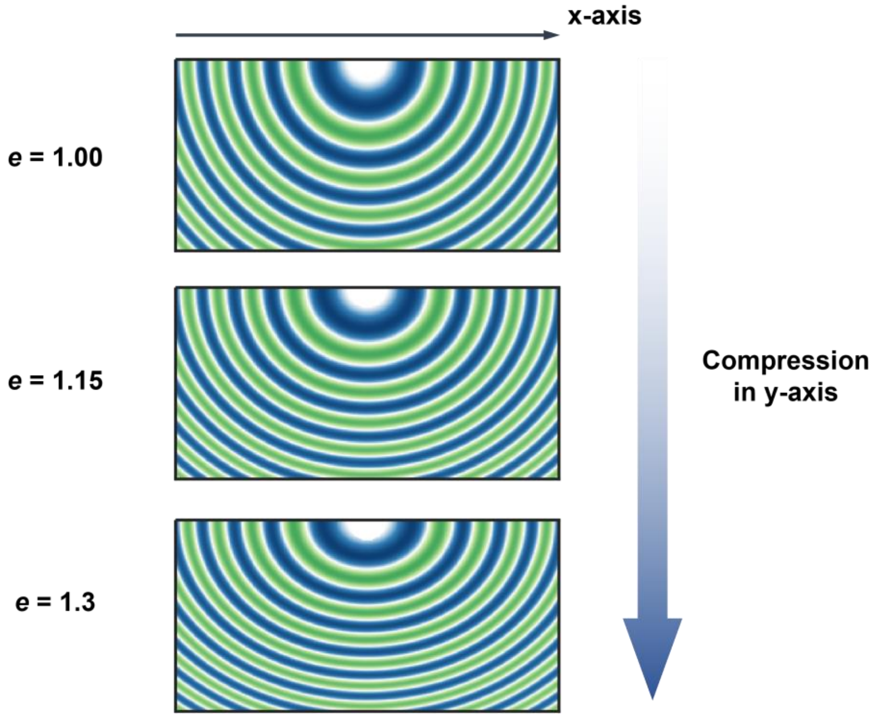


**Fig. S4.** Schematic diagram of y-axis compression of SAFWB

As demonstrated by Equation S2 in the Supplementary Material, the introduced compressibility factor e allows for independent control over the beam's orientation along the y-axis. This effect is attributed to the compression of the SAFWB function in the y-dimension induced by factor e.

$$W_y = e^{-a(y\cdot e)} \sin(\frac{4}{3} b^{\frac{1}{2}} k (y \cdot e)^{\frac{3}{2}}) \frac{1}{g}. \tag{S2}$$

## Supplementary Note 5. Experimental and theoretical simulation results of self-accelerating and self-healing characteristics of SAFWB

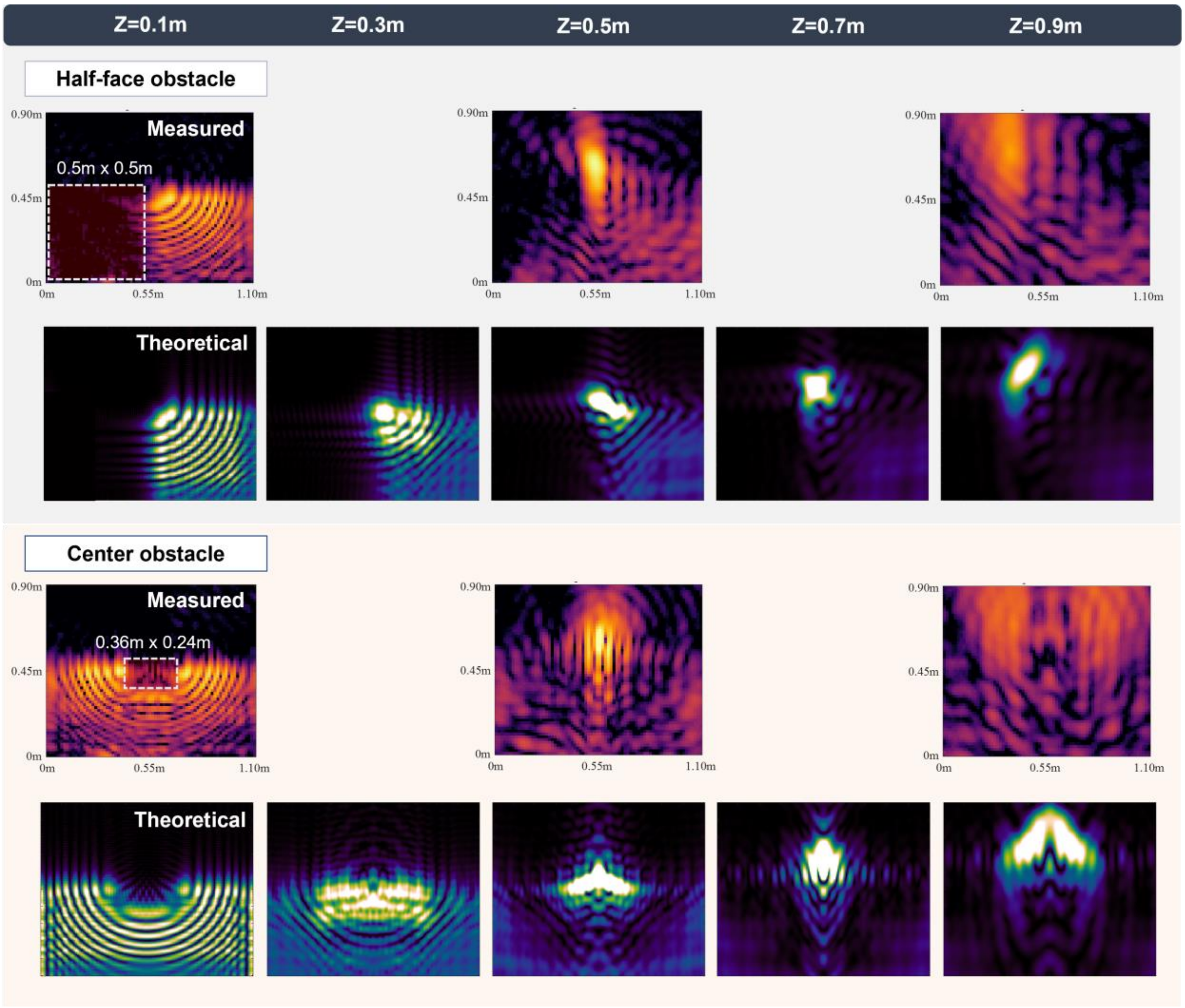


**Fig. S5.** Experimental and theoretical simulation results of self-accelerating and self-healing characteristics of SAFWB.

As detailed below, we have now incorporated the corresponding theoretical simulation results for the experimentally measured self-accelerating and self-healing behaviors, as show in Fig. S5. Under the partial beam blocking scenario with a half-face obstacle, the experimentally measured result is presented in the first row of Fig. S5. As the propagation distance z increases, the beam energy on the lower-right side shifts towards the upper-left side due to the self-accelerating property. The second row shows the corresponding theoretical simulation result, which we have newly supplemented. This result was calculated based on the Rayleigh-Sommerfeld diffraction integral formula. Both the experimental and theoretical results show a

consistent trend in the energy focal spot movement. However, the measured results exhibit a noticeable energy diffusion. This discrepancy is attributed to the fact that the actual excitation wave is a spherical wave rather than an ideal plane wave. Similarly, in the case of a center obstacle, the trend of energy shifting also remains consistent. However, the experimentally measured energy distribution appears more dispersed in this scenario. Nevertheless, the measured and theoretical simulation results exhibit high consistency at short propagation distances.

## Supplementary Note 6. Comparison between Airy beam and Weber beam

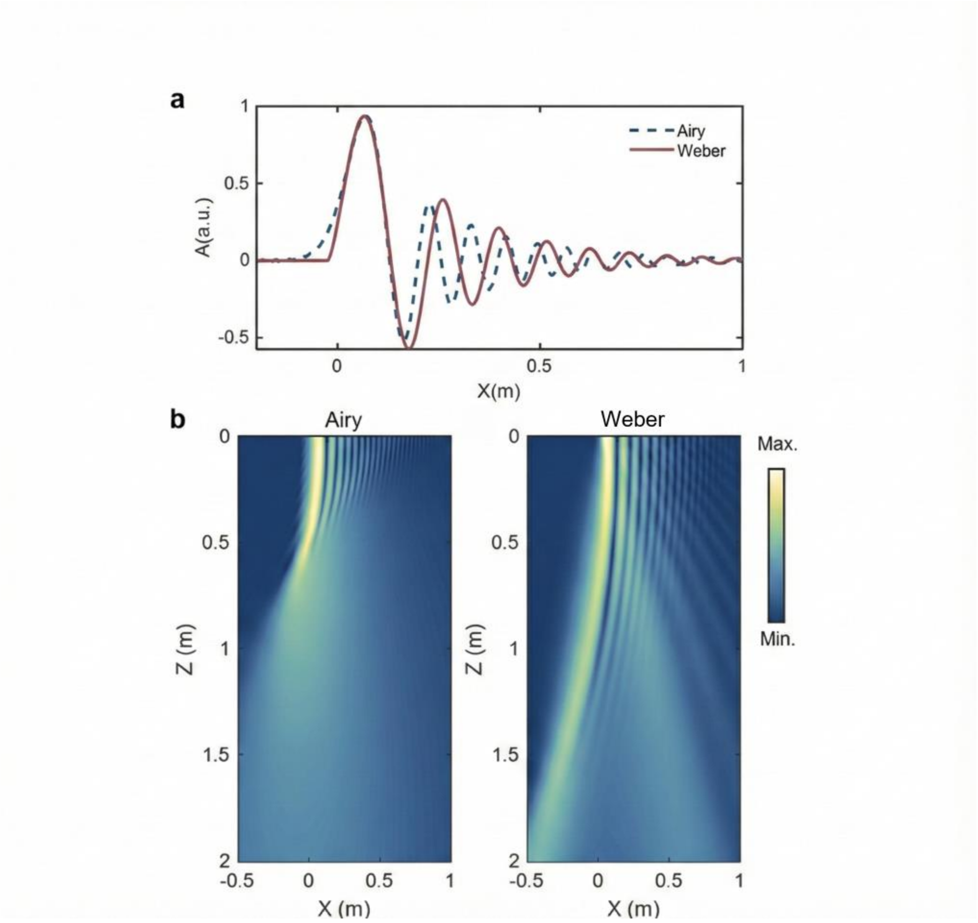


**Fig. S6.** Comparison between Airy and Weber beams. (a) Amplitude distribution at the propagation direction z = 0 for both beams. (b) Propagation of the Airy beam and Weber beam within the plane.

We agree that the family of non-diffracting beams has indeed seen the birth and development of numerous beam types. The Airy and Bessel beams stand as typical representatives that have received widespread research and application. To address your question concerning our motivation for employing the Weber beam, we provide an answer through a direct comparison between the Airy and Weber beams.

As shown in Fig. S6a, we set Airy wave packets and Weber wave packets with similar energy. The Airy function is shown in Equation S3:

$$Airy(x,z)=\frac{1}{\pi}\int_0^{\infty}\cos\{\frac{1}{3}t^3+[S(x)-\frac{\xi^2(z)}{4}+ja_1\xi(z)]\cdot t\}dt\cdot e^{[a_1S(x)-\frac{a_1}{2}\xi^2(z)-j\frac{1}{12}\xi^3(z)+j\frac{a^2}{2}\xi(z)+j\frac{1}{2}S(x)\xi(z)]}. \quad \text{(S3)}$$

The Weber function is characterized based on Equation 1, with parameters a = 4.5, b = 0.1, and g = 0.7. As illustrated in Fig. S6b, under a similar initial energy distribution, the Airy beam

maintains its non-diffracting state only within 0.5 m. In contrast, the Weber beam is capable of propagating to distances exceeding 1.5 m while remaining in the non-diffracting state. Therefore, in order to maximize the energy utilization, we adopted the Weber beam scheme in this work.

## Supplementary Note 7. Comparison of theoretical simulation and measured phase results of OWB

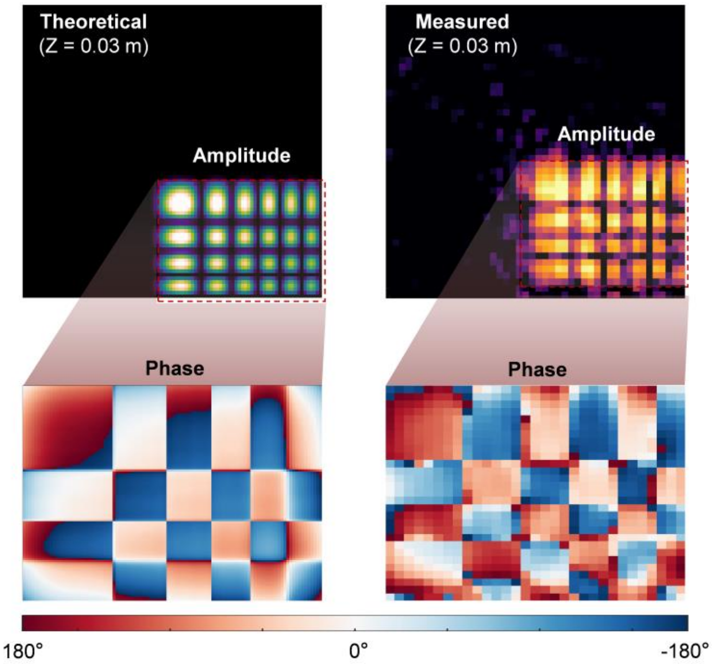


**Fig. S7.** Comparison of theoretical simulation and measured phase results of OWB.

As shown in Fig. S7, the comparative distributions of amplitude and phase at a distance of 0.03 m from the metasurface are provided. The measured results clearly reveal a distinct phase difference between the different beams, exhibiting a 180° shift. Moreover, the measured phase distribution aligns well with the theoretical results. The 180° phase difference between the beams enables their non-diffracting characteristics.

# Supplementary Note 8. Interpolation fitting process

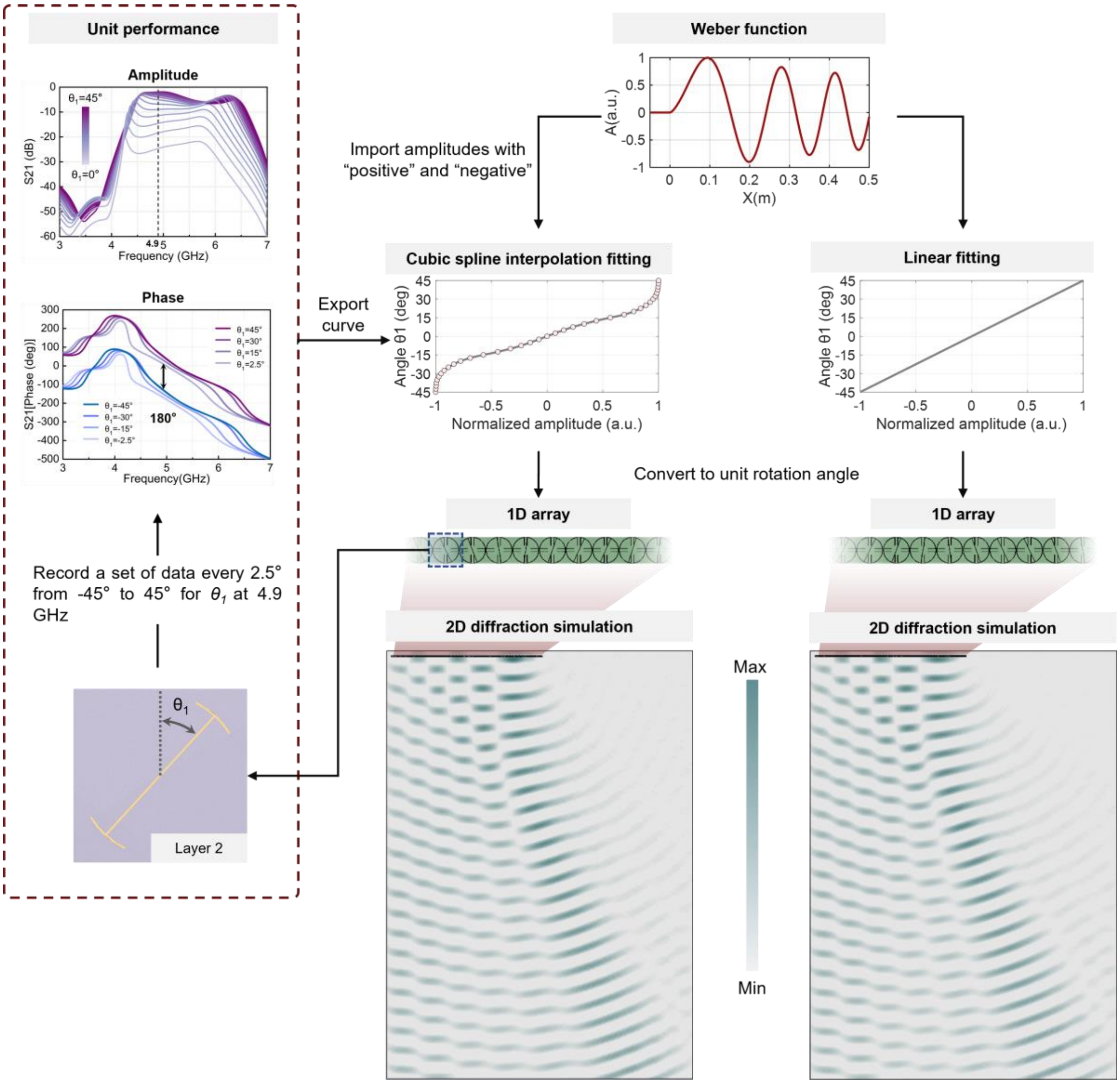


**Fig. S8.** Metasurface arrangement workflow and a comparison of different fitting approaches on beamforming performance.

Regarding the sensitivity of beam quality to the errors in $\theta_1$ arising during the fitting or fabrication processes, we have performed a comparative simulation. Specifically, we analyzed the diffraction results of 1D Weber arrays generated by different fitting methods within CST Studio Suite, as illustrated in Fig. S8. In order to deliberately introduce substantial error in $\theta_1$, we utilized linear fitting as a substitute for cubic spline interpolation in our control group. To our surprise, even with such a pronounced difference between the fitting profiles, the resulting 2D diffraction distributions remained highly consistent. This outcome demonstrates that the proposed Weber beam is inherently robust to localized variations or minor inaccuracies in the rotation angle $\theta_1$.

# Supplementary Note 9. Relative bandwidth

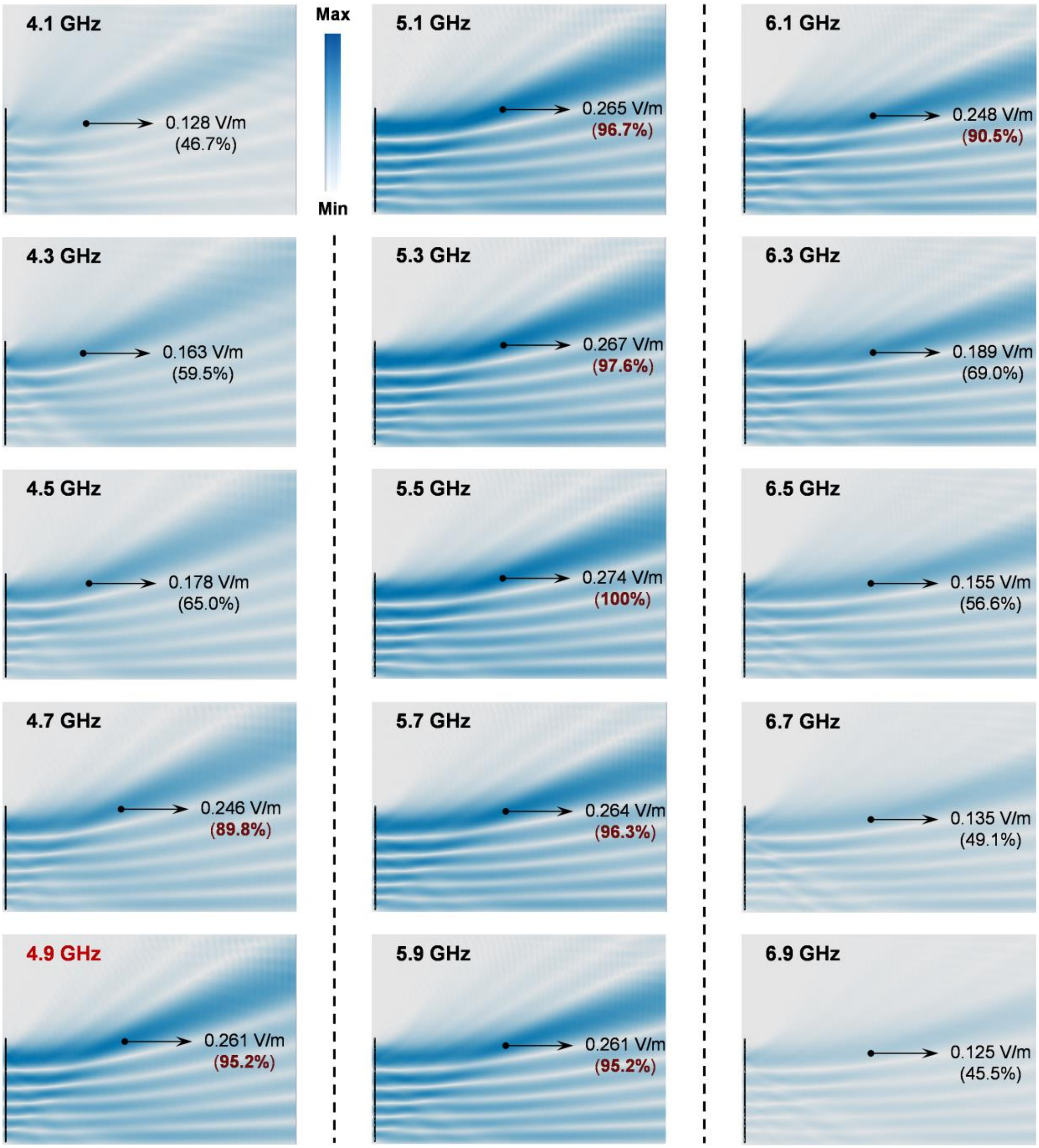


**Fig. S9.** The propagation cross-section of one-dimensional Weber beams at different frequencies.

We have supplemented the work with simulations to illustrate the performance of the proposed beam across the realistic communication bandwidth. Given that OWB and SAFWB are both composed of 1D Weber functions, we utilized the transverse 1D component within the OWB for our additional analysis. As shown in Fig. S9, we simulated this 1D Weber function across a frequency sweep from 4.1 to 6.9 GHz with a fine step, aiming to characterize the evolution of

the longitudinal energy profile over the operating bandwidth. As indicated by the unit cell response in Fig. 3c, the PB phase control remains functional from 4.5 to 6 GHz. To address this, we conducted wideband simulations with a discrete frequency sweep in CST software to obtain the spatial energy profiles for each frequency, as shown in Fig. S9. The maximum field strengths at the beam curvature were measured across the spectrum, using the intensity at 5.5 GHz as a reference (100%). By setting a 90% power-level criterion for operational effectiveness, the simulations reveal that the beam maintains over 90% of its peak energy within the 4.7–6.1 GHz range. Therefore, the operational bandwidth of the proposed Weber beam is identified as 4.7–6.1 GHz, yielding a relative bandwidth of 25.9%.

## Supplementary Note 10. Multi-user communication scenario

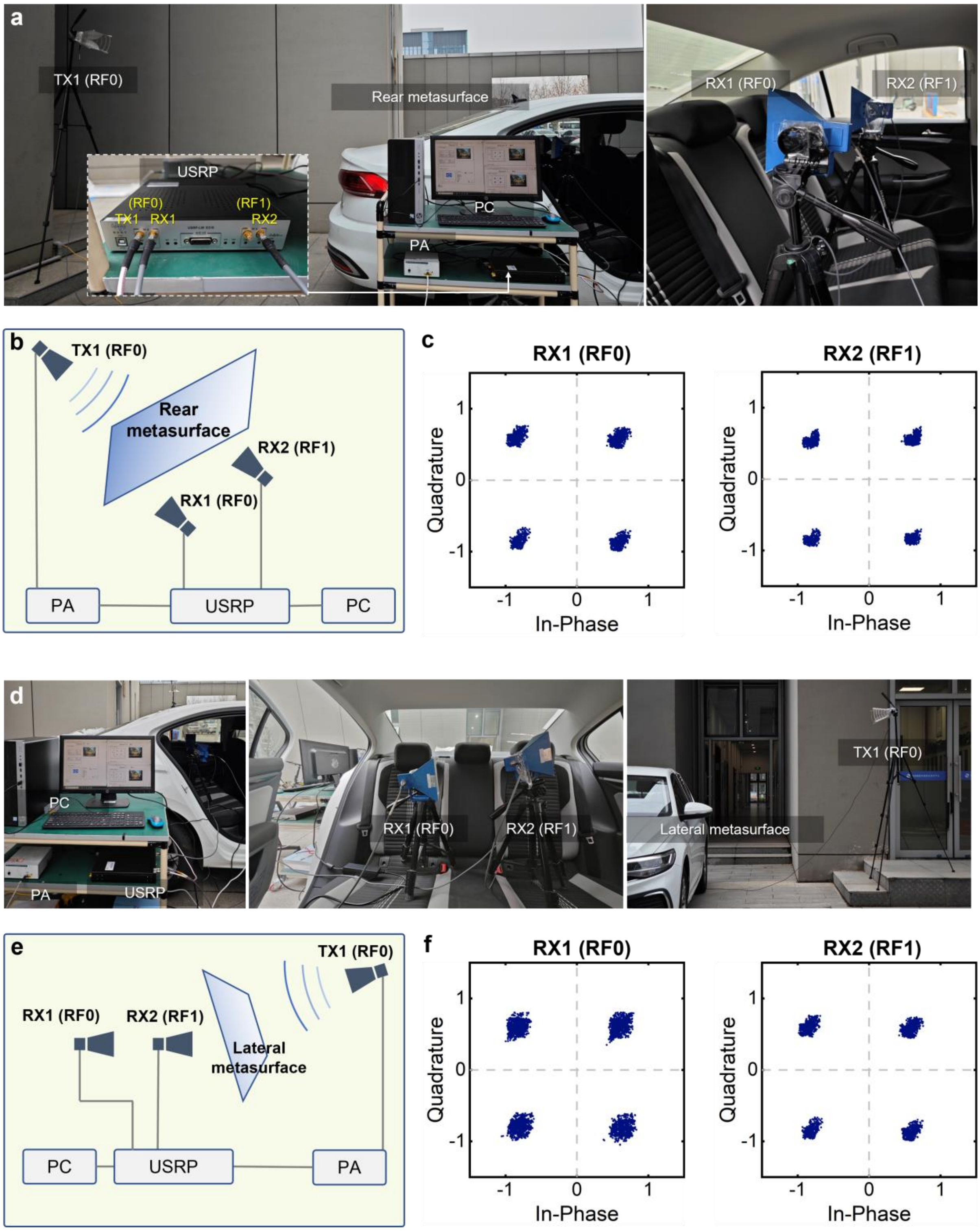


**Fig. S10.** Multi-user communication scenario.

A realistic vehicle cabin setup was established to validate the multi-user/multi-path communication capabilities of the metasurface-enhanced window. We expanded the hardware configuration by adding a second receiving link to the USRP to support dual-antenna testing. Following the configuration in Figs. S10a-c, the rear-window metasurface was excited by an

external transmitting antenna. Internally, two receivers (connected to USRP ports RF0 and RF1) were located in the primary lobe areas following the SAFWB's focus, as defined in Fig. S3.1b. By utilizing two horizontally crossed Weber beams, the system successfully covers the left, center, and right seating positions, facilitating multi-path communication. The measured constellation diagrams confirm high-fidelity data transmission at both receiver locations.

For the lateral-window investigation, a similar hardware configuration was employed, using an external excitation source and two internal receivers, as shown in Fig. S10d-f. Unlike the SAFWB configuration, the lateral-window OWB leverages its inherent self-healing capability to maintain a robust multi-user link despite physical blockage. According to the field distributions in Fig. 4c, the OWB main lobe exhibits the ability to recover by utilizing energy from its adjacent sidelobes. To test this, two receivers were placed in-line along the beam's trajectory. Although RX2 is positioned in the obstructed path behind RX1 and relies on the reconsolidated sidelobe energy, it still achieves successful data transmission. Although RX2's constellation quality shows some degradation compared to RX1, both receivers exhibit legible and high-fidelity constellations.

## Supplementary Note 11. Communication scenario with passenger

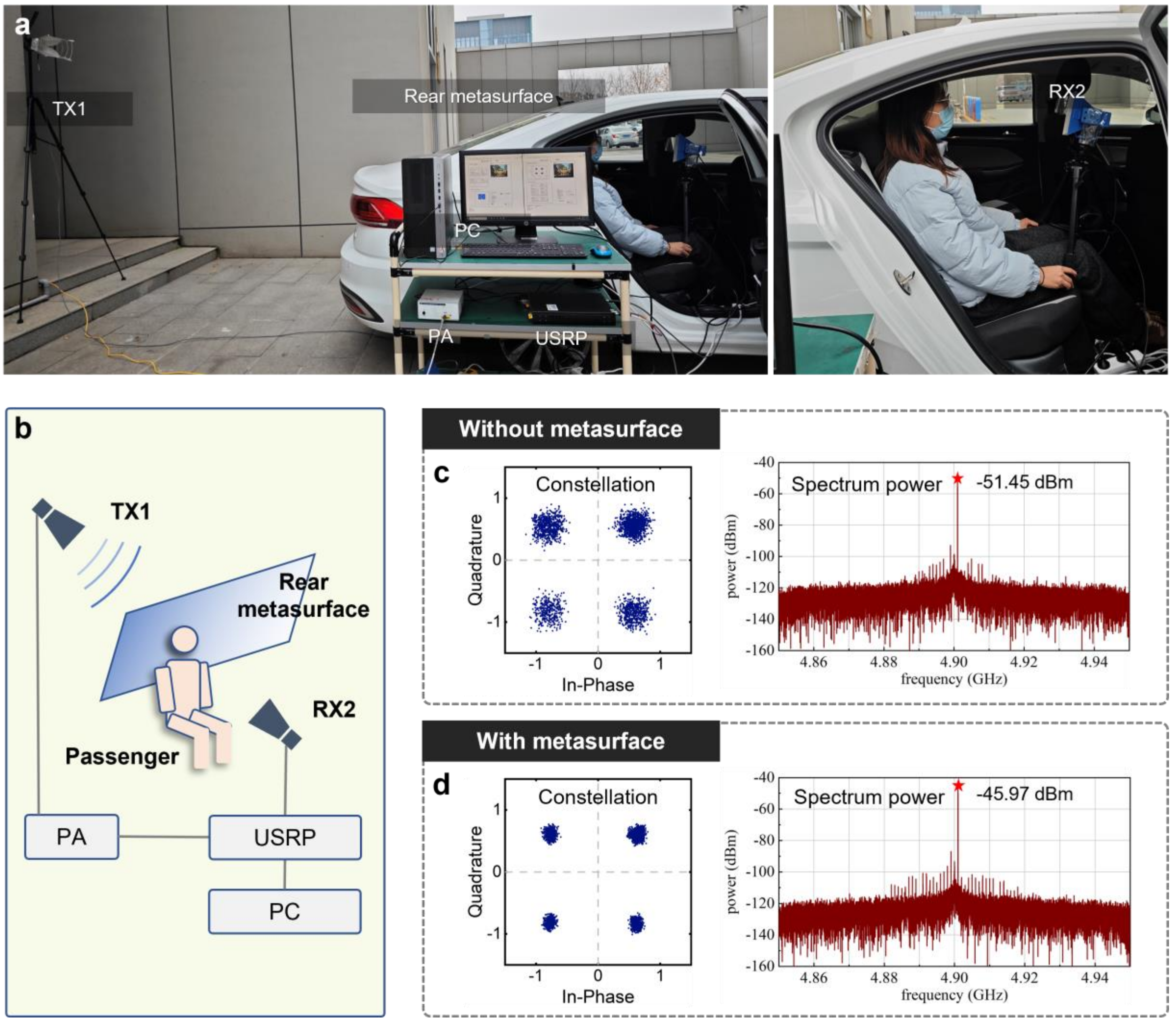


**Fig. S11. 1.** Rear metasurface communication scenario with passenger.

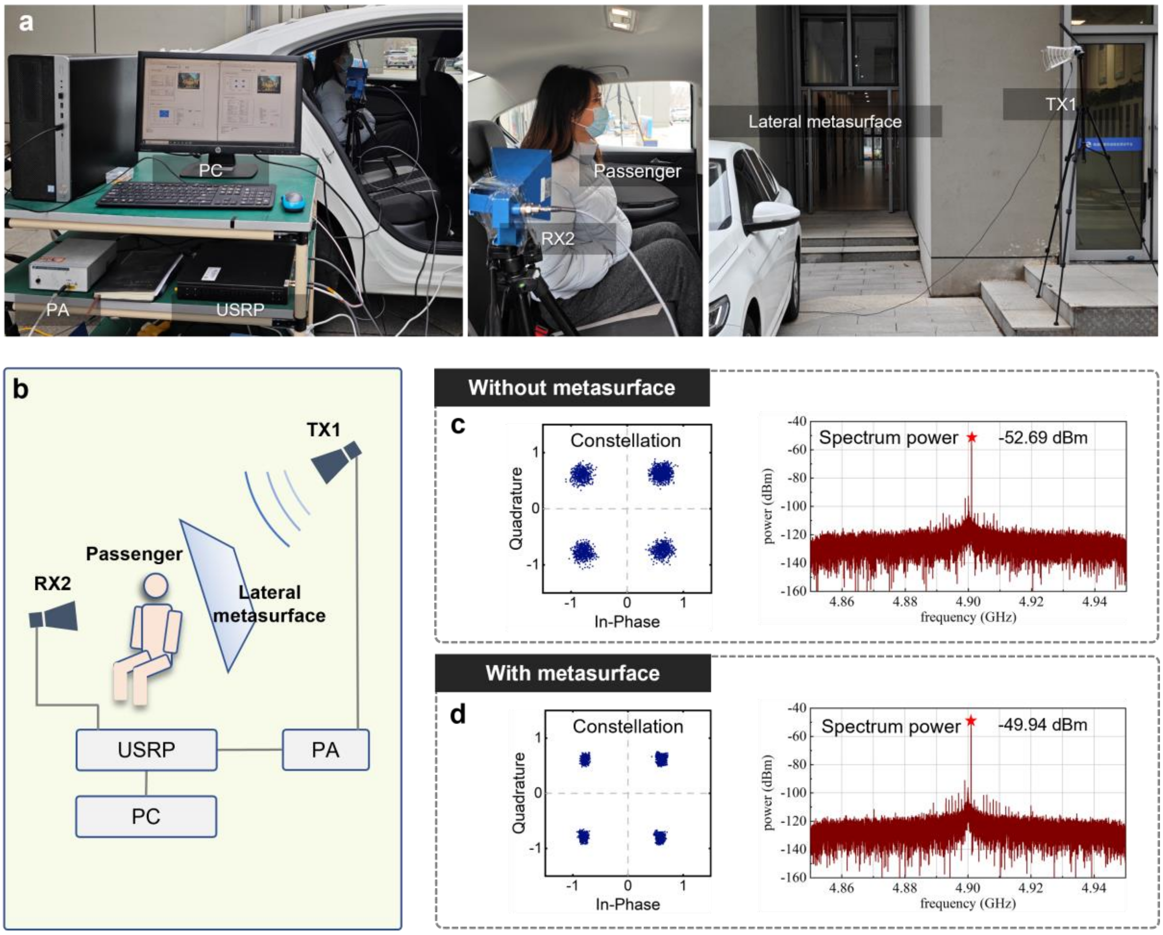


**Fig. S11. 2.** Lateral metasurface communication scenario with passenger.

To provide a more comprehensive representation of the in-cabin communication scenarios, we have conducted additional experiments involving the presence of passengers for both the rear and lateral windows, as illustrated in Figs. S11.1 and 2. In the rear-window scenario shown in Fig. S11.1, the passenger acts as an obstruction positioned between the metasurface and the receiving antenna RX2. The Weber beam generated by the metasurface effectively bypasses the passenger, directing more energy into the core region in front of them. We compared the cases with and without the metasurface; the results with the metasurface exhibit significantly higher-quality constellation diagrams and increased received signal power. Similarly, for the side-window setup in Fig. S11.2, where the passenger sits adjacent to the metasurface and the receiver RX2 is on the far side, the system's performance remains robust. The cases integrated with metasurface show marked improvements in both constellation clarity and signal strength, further validating the effectiveness of the Weber beam in complex in-cabin environments.

## Supplementary Note 12. The impact of obstacle size on communication.

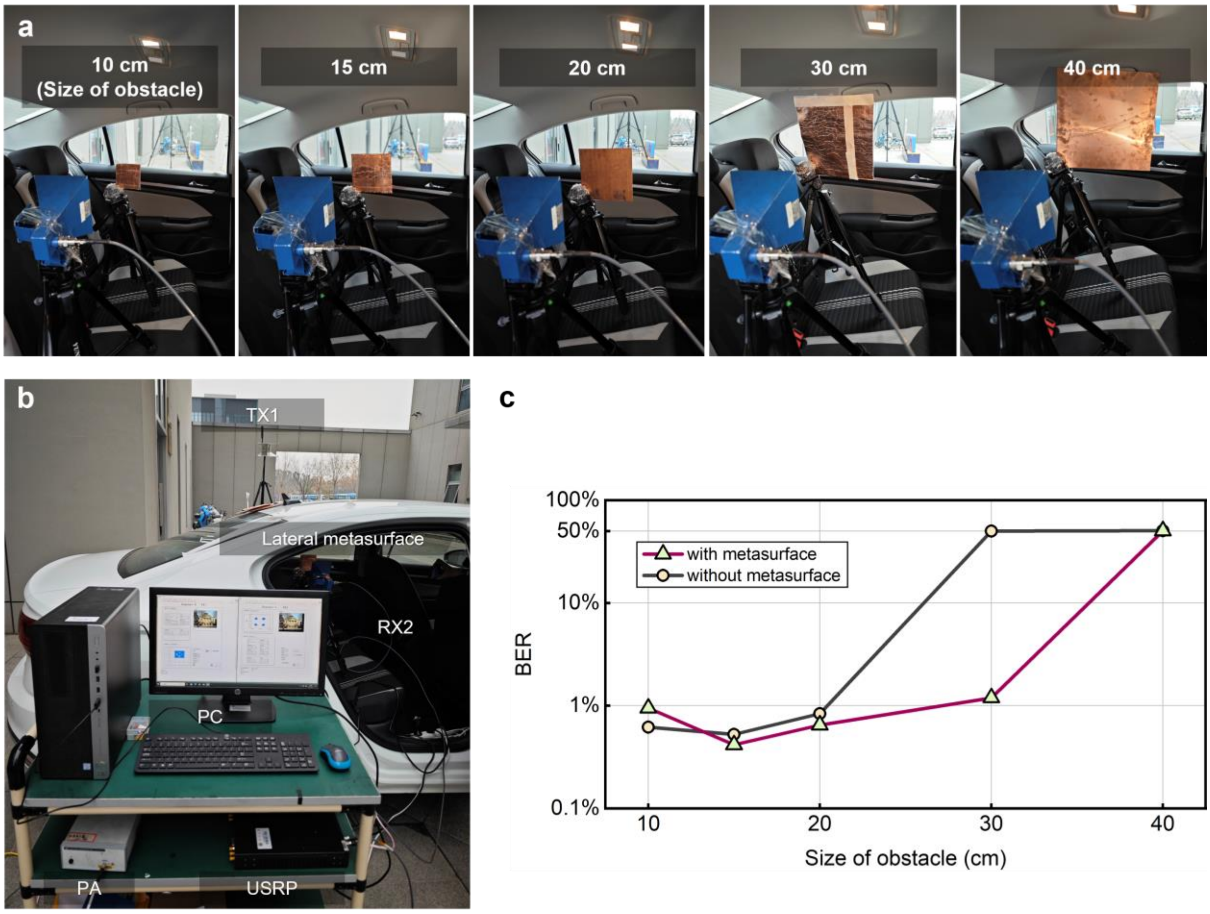


**Fig. S12.** The impact of obstacle size on communication.

To evaluate the robustness threshold, square metallic blocks were placed in the transmission path. Without the metasurface, the communication link broke down once the obstacle size reached 30 cm. However, with the metasurface, the link remained robust, with the breakdown point extended to 40 cm. Consequently, the OWB from the Lateral window exhibits a tolerance threshold of 30 cm for interior obstacles. This is consistent with our rear-window findings, which indicate a capability to bypass obstructions up to half the window's aperture.
